# Metabolic Reprogramming Supports Neonicotinoid Resistance in the Brown Planthopper, *Nilaparvata lugens*

**DOI:** 10.3390/insects17090884

**Published:** 2026-08-24

**Authors:** Guijian Zhang, Minghao Jiang, Xiangqian Chang, Liang Lv

**Affiliations:** 1 Hubei Key Laboratory for Crop Diseases & Insect Pests & Weeds Control, Key Laboratory of Integrated Pests Management on Crops in Central China of Ministry of Agricultural and Rural Affairs, Institute of Plant Protection and Soil Science, Hubei Academy of Agricultural Sciences, Wuhan 430064, China; 2 Hubei Insect Resources Utilization and Sustainable Pest Management Key Laboratory, College of Plant Science and Technology, Huazhong Agricultural University, Wuhan 430070, China

**Keywords:** brown planthopper, neonicotinoid resistance, metabolic reprogramming, isocitrate dehydrogenase, UDP-glycosyltransferase

## Abstract

Metabolic resistance is commonly attributed to the overexpression of detoxification enzymes, whereas the metabolic systems that sustain detoxification remain less well resolved. Here, we integrated widely targeted metabolomics and transcriptomics to compare the clothianidin-resistant brown planthopper strain CLR with the susceptible strain CLS and validated candidate genes in an independent nitenpyram-resistant background. CLR and CLS exhibited distinct metabolomic profiles. Differential genes and metabolites converged on carbon metabolism, glycolysis/gluconeogenesis, the tricarboxylic acid cycle, glutathione metabolism, pentose and glucuronate interconversions, cytochrome P450-mediated xenobiotic metabolism, and ABC transporters. These changes were summarized into four interconnected modules involving glycolytic energy supply, protective sugar-derived metabolites, pyruvate–TCA–malate metabolism, and UGT-mediated glycosylation. Expression analysis identified a mitochondrial NADP-dependent isocitrate dehydrogenase gene and UDP-glucosyltransferase 2 (UGT2) as consistently upregulated in both resistant backgrounds. Silencing *NADP* reduced the LC_50_ of nitenpyram and clothianidin by 1.84- and approximately 1.81-fold, respectively, whereas *UGT2* silencing produced corresponding reductions of 1.89- and 1.83-fold. These findings indicate that neonicotinoid resistance in *Nilaparvata lugens* is supported by coordinated remodelling of central metabolism and detoxification and identify two candidate metabolic nodes for further resistance management research.

## Simple Summary

Insecticide resistance makes the brown planthopper, *Nilaparvata lugens*, increasingly difficult to control. Resistance is often linked to enzymes that break down insecticides, but these enzymes also depend on energy and other metabolites produced by the insect. In this study, we compared insecticide-resistant and susceptible brown planthoppers to investigate how metabolism contributes to resistance. Resistant insects showed coordinated changes in pathways related to energy production, protective metabolites, and insecticide detoxification. Two genes, mitochondrial NADP-dependent isocitrate dehydrogenase (NADP) and UDP-glucosyltransferase 2 (UGT2), were consistently more active in two independently selected resistant strains. Silencing either gene increased the insects’ sensitivity to the neonicotinoid insecticides nitenpyram and clothianidin. These results suggest that neonicotinoid resistance is supported by interconnected changes in both central metabolism and detoxification. NADP and UGT2 may therefore represent useful molecular targets for monitoring resistance and developing improved pest-management strategies.

## 1. Introduction

The brown planthopper, *Nilaparvata lugens* Stål, is one of the most destructive migratory pests of rice in Asia, where it damages plants through sustained phloem feeding, causes hopperburn during outbreaks and transmits rice viruses [1]. Its high fecundity, long-distance migration and rapid population growth make chemical control an important component of emergency management in many rice-growing regions [1]. Neonicotinoid insecticides have been widely used against *N. lugens* and other sap-feeding pests because they act mainly on insect nicotinic acetylcholine receptors and show strong systemic activity in plants [2,3,4,5]. Nevertheless, intensive and repeated use of neonicotinoids has accelerated resistance evolution in many insect pests, reducing field efficacy and increasing the need for resistance management strategies [6,7,8]. Understanding the mechanisms underlying neonicotinoid resistance in *N. lugens* is therefore important for improving insecticide stewardship and delaying resistance development.

Insecticide resistance is usually a multifactorial phenotype involving altered target-site sensitivity, reduced penetration, behavioural avoidance and enhanced metabolic detoxification [6,7,9,10]. Among these mechanisms, metabolic resistance has received particular attention because it can reduce the internal dose of insecticides before they reach their molecular targets [9,10]. In *N. lugens*, overexpression of the cytochrome P450 gene *CYP6ER1* has been associated with imidacloprid resistance, providing direct evidence that P450-mediated detoxification contributes to neonicotinoid resistance in this species [11]. Additional studies and reviews have emphasized that P450s, glutathione S-transferases (GSTs), carboxylesterases, UDP-glycosyltransferases and ATP-binding cassette (ABC) transporters may contribute to insecticide resistance through altered expression, substrate metabolism, conjugation or transport [9,11,12,13,14]. For example, GSTs participate in glutathione-dependent conjugation and oxidative-stress defence, whereas ABC transporters can export xenobiotics or their metabolites across membranes [12,13,15]. These studies have greatly advanced our understanding of detoxification-based resistance, but they have generally focused on detoxification genes themselves rather than on the metabolic conditions that allow these reactions to be sustained.

From a biochemical perspective, detoxification is not an isolated enzymatic process. P450-catalysed oxidation requires electron transfer from NADPH through NADPH-cytochrome P450 reductase, GST-mediated conjugation depends on reduced glutathione, and UGT-mediated glycosylation requires activated UDP-sugars as donor substrates [13,14,16]. Detoxification products may also need to be transported across membranes through ATP-dependent transport systems [12,15]. Thus, enhanced detoxification capacity is likely to impose demands on central metabolism, especially pathways that generate ATP, *NADPH*, glutathione and sugar donors [16,17,18,19]. This requirement is consistent with the classical framework of xenobiotic metabolism, in which phase I oxidation, phase II conjugation and phase III transport are functionally connected to cellular energy, redox balance and precursor supply [9,12,16,20]. Therefore, insecticide resistance may involve not only the up-regulation of detoxification enzymes, but also the reallocation of metabolic fluxes that supply cofactors and substrates for detoxification reactions.

Evidence from other insects supports this view. In *Drosophila melanogaster*, Cyp6g1 overexpression is associated with dichlorodiphenyltrichloroethane (DDT) resistance and has become a representative example linking a P450 gene to insecticide resistance in a model insect [21,22]. In *Anopheles gambiae*, CYP6M2 and CYP6P3 are elevated in pyrethroid-resistant populations, and CYP6M2 can metabolize pyrethroids directly [23,24]. In the whitefly *Bemisia tabaci*, overexpression of CYP6CM1 is associated with resistance to neonicotinoid insecticides, indicating that P450-mediated detoxification is also important in sap-feeding hemipterans [25]. These examples show that detoxification enzymes can be causal contributors to resistance, but they also imply a continuous requirement for cofactors, reducing equivalents and metabolic substrates. Similar principles have been described in biomedical studies, where drug-tolerant cells frequently exhibit altered glucose metabolism, pentose phosphate pathway activity, *NADPH* production and glutathione homeostasis [18]. In such systems, pathways that maintain redox balance can influence the capacity of cells to tolerate xenobiotic or oxidative stress [17,19]. These observations suggest that tolerance to toxic compounds is closely linked to cellular metabolic state across diverse biological systems.

For insecticide resistance, this metabolic dimension deserves closer attention. After insecticides enter the insect body, they not only require metabolic conversion by P450s, GSTs, UGTs and related enzyme systems, but also may induce oxidative stress, increase energy demand and disturb cellular homeostasis [9,10,16]. If resistant insects enhance glycolysis, the tricarboxylic acid cycle, the pentose phosphate pathway, amino acid metabolism or glycosylation-related pathways to provide ATP, NADPH, glutathione and UDP-sugars, these metabolic processes may contribute to the maintenance of detoxification capacity and resistance phenotypes [16,17,18,19]. Recent transcriptomic, metabolomic and multi-omics approaches provide opportunities to examine such coordinated changes at the pathway level rather than only at the level of individual detoxification genes [26,27,28]. However, it remains unclear whether metabolic network changes in neonicotinoid-resistant *N. lugens* are merely associated responses to insecticide stress or whether they actively participate in resistance maintenance.

In this study, we investigated the potential contribution of metabolic regulation to neonicotinoid resistance in *N. lugens*. By integrating transcriptomic and metabolomic analyses with candidate-gene expression validation and RNA interference, we aimed to evaluate whether specific metabolic processes participate in resistance maintenance and may provide potential targets for resistance management or synergistic control strategies.

## 2. Materials and Methods

### 2.1. Insect Strains, Rearing and Sample Collection

The clothianidin-resistant (CLR) and corresponding susceptible (CLS) strains were obtained from laboratory colonies previously established and characterized by Jin et al. [29], whereas the nitenpyram-resistant (NR) and corresponding susceptible (NS) strains were established as described by Mao et al. [30]. The CLR and NR strains had been maintained under continuous selection with the corresponding insecticides for more than 20 generations, while the susceptible strains were maintained without insecticide exposure. Before the experiments, resistance phenotypes were reconfirmed using the rice-seedling dipping bioassay described in Section 2.6. Based on the estimated LC_50_ values, the CLR and NR strains exhibited approximately 270-fold and 100-fold resistance, respectively, relative to their corresponding susceptible strains. Taichung Native 1 (TN1) rice seedlings were used for insect rearing and bioassays following a previously reported procedure [31]. The insects were maintained at 27 ± 1 °C, 70–80% relative humidity, and a photoperiod of 16 h light:8 h dark. Water was replenished as required to maintain seedling growth, and no additional fertilizer was applied during the experimental period. Fourth-instar nymphs were selected with reference to previous resistance-related studies in *N. lugens* [29,30]. The use of a single late-nymphal stage minimized developmental variation, provided sufficient biomass for metabolomic and transcriptomic analyses, and facilitated standardized microinjection. Adults were not used because differences in sex, reproductive status, and wing morph could introduce additional physiological and metabolic variation.

For metabolomic analysis, each strain contained six biological replicates. Each biological replicate consisted of 100 mg of fourth-instar nymphs. For transcriptome sequencing, each strain contained three biological replicates, with each biological replicate consisting of 20 fourth-instar nymphs. Samples were immediately frozen in liquid nitrogen after collection and stored at −80 °C until metabolite or RNA extraction.

### 2.2. Metabolite Profiling and Data Analysis

Metabolite extraction and profiling were performed by Biomarker Technologies Corporation (Beijing, China) following a previously published workflow [32]. Six biological replicates were analyzed for each strain, and each replicate consisted of 100 mg of pooled fourth-instar nymphs. Frozen samples were ground in liquid nitrogen and subjected to metabolite extraction. Equal aliquots of all extracts were pooled to prepare quality-control (QC) samples, which were analyzed periodically throughout the analytical sequence to monitor instrumental stability and analytical repeatability.

Metabolite profiling was performed using a Shimadzu Nexera X2 UPLC system (Shimadzu Corporation, Kyoto, Japan) coupled to an AB Sciex 4500 QTRAP mass spectrometer (AB SCIEX, Framingham, MA, USA) equipped with an electrospray ionization source. Chromatographic separation was achieved using an Agilent SB-C18 column (Agilent SB-C18 column) (1.8 μm, 2.1 × 100 mm) maintained at 40 °C. The mobile phases consisted of water containing 0.1% formic acid (A) and acetonitrile containing 0.1% formic acid (B). The proportion of B was increased from 5% to 95% within 9 min, maintained at 95%, returned to 5% within 1.1 min, and equilibrated for 2.9 min. The injection volume was 4 μL. The mass spectrometer was operated in positive- and negative-ion modes with a source temperature of 550 °C and ion-spray voltages of 5500 and −4500 V, respectively. Metabolites were detected in multiple reaction monitoring (MRM) mode and identified by comparison of precursor/product ions and MS/MS spectra with the Biomarker Technologies in-house database and the HMDB, MassBank, MoToDB, and METLIN databases [32]. Relative quantification was based on integrated and corrected MRM peak areas.

Principal coordinate analysis (PCoA) was used to evaluate overall differences between the clothianidin-resistant (CLR) and susceptible (CLS) strains. Orthogonal partial least-squares discriminant analysis was performed using SIMCA-P version 14.0, and variable importance in projection (VIP) values were obtained from the resulting model. Metabolites with a fold change of ≥2.0 or ≤0.5 and a VIP value of ≥1.0 were considered differentially abundant. Differential metabolites were annotated against the KEGG database and subjected to pathway-enrichment analysis. For each pathway, the differential-abundance score was calculated as (Nup − Ndown)/(Nup + Ndown), where Nup and Ndown represent the numbers of metabolites with higher and lower abundance, respectively, in CLR relative to CLS.

### 2.3. Transcriptome Sequencing, Integrated Pathway Analysis and Candidate Gene Screening

Total RNA was extracted from whole insects using TRIzol reagent according to the manufacturer’s instructions. RNA concentration and integrity were assessed using a spectrophotometer and an Agilent 2100 Bioanalyzer (Agilent Technologies, Santa Clara, CA, USA), and only high-quality RNA samples were used for library construction. Transcriptome libraries were sequenced on an Illumina platform (Illumina, San Diego, CA, USA), and raw reads were filtered to obtain clean reads. Clean reads were aligned to the *N. lugens* reference genome (NCBI assembly GCA_000757685.1, BioProject PRJNA177647) for gene annotation and quantification.

Differentially expressed genes (DEGs) between resistant and susceptible strains were identified using DESeq2 with thresholds of |log_2_ fold change | ≥ 1 and false discovery rate (FDR) < 0.05. KEGG enrichment analysis was then performed to identify significantly affected biological pathways. To integrate transcriptomic and metabolomic information, DEGs and differential metabolites were jointly mapped to KEGG pathways, and pathways containing both altered genes and altered metabolites were considered key candidate pathways. On this basis, candidate genes involved in carbon metabolism, glycolysis/gluconeogenesis, fructose and mannose metabolism, pentose and glucuronate interconversions, pyruvate metabolism, the citrate cycle, and related pathways were selected for further validation.

### 2.4. qRT-PCR Validation

Total RNA from susceptible and resistant strains was extracted using TRIzol reagent (Invitrogen, Carlsbad, CA, USA). One microgram of total RNA was reverse-transcribed into first-strand cDNA using the PrimeScript RT Reagent Kit (Takara, Shiga, Japan). qRT-PCR was performed on a QuantStudio 5 Real-Time PCR System (Applied Biosystems, Waltham, MA, USA) using SYBR Green Master Mix (Applied Biosystems, Waltham, MA, USA). Each 10 uL reaction contained cDNA template, forward and reverse primers, and SYBR Green reaction mix according to the manufacturer’s recommendations.

The thermal cycling program consisted of an initial denaturation at 95 degrees C for 10 min, followed by 40 cycles of 95 degrees C for 15 s and 60 degrees C for 1 min. Expression levels of target genes were normalized against the reference genes 18S rRNA and beta-actin, and relative expression levels were calculated using the 2^−ΔΔCt^ method [33]. Each treatment included three biological replicates and three technical replicates. Primers used for qRT-PCR are listed in Table S1.

### 2.5. RNA Interference

For functional validation, double-stranded RNA (dsRNA) targeting candidate genes was synthesized using the T7 RiboMAX Express RNAi System (Promega, Madison, WI, USA) according to the manufacturer’s instructions. dsEGFP was used as the negative control. Approximately 50 nL of dsRNA solution at 1000 ng/μL was microinjected into the membranous region between the prothorax and mesothorax of fourth-instar nymphs using a microprocessor-controlled Nanoliter 2020 injector (World Precision Instruments, Sarasota, FL, USA), following a previously reported RNAi procedure for *N. lugens* [34]. Injected insects were maintained under standard rearing conditions for 24 h, and dead or visibly damaged individuals were excluded from subsequent analysis. Knockdown efficiency was confirmed by qRT-PCR.

### 2.6. Insecticide Bioassays

Bioassays were conducted using the rice-stem dipping method. Rice stems were immersed in the insecticide solutions for 30 s, air-dried at room temperature, and transferred to assay cups. Candidate gene-silenced insects were then placed on the treated rice stems. Each treatment included three biological replicates of 15 insects each, and mortality was recorded 96 h after exposure. For the RNAi assays, dsEGFP-treated insects served as the control. The tested concentrations were 0, 20, 40, 80, 160, and 320 mg/L for nitenpyram and 0, 60, 120, 240, 480, and 960 mg/L for clothianidin.

### 2.7. Statistical Analysis

qRT-PCR expression data and bioassay data are presented as the mean ± SEM. Differences in relative gene expression between two groups were analyzed using Student’s *t*-test. Relative gene expression levels were calculated using the 2^−ΔΔCt^ method prior to statistical analysis. General statistical analyses and graph preparation were performed using GraphPad Prism 9.0 (GraphPad Software, San Diego, CA, USA). Mortality data were corrected using Abbott’s formula when necessary, and LC_50_ values and their 95% confidence intervals were estimated by probit analysis using PoloPlus 2.0 (LeOra Software, Berkeley, CA, USA). *p* < 0.05 was considered statistically significant.

## 3. Results

### 3.1. The Resistant Strain Exhibits a Distinct Metabolomic Profile Relative to the Susceptible Strain

**Figure 1:**
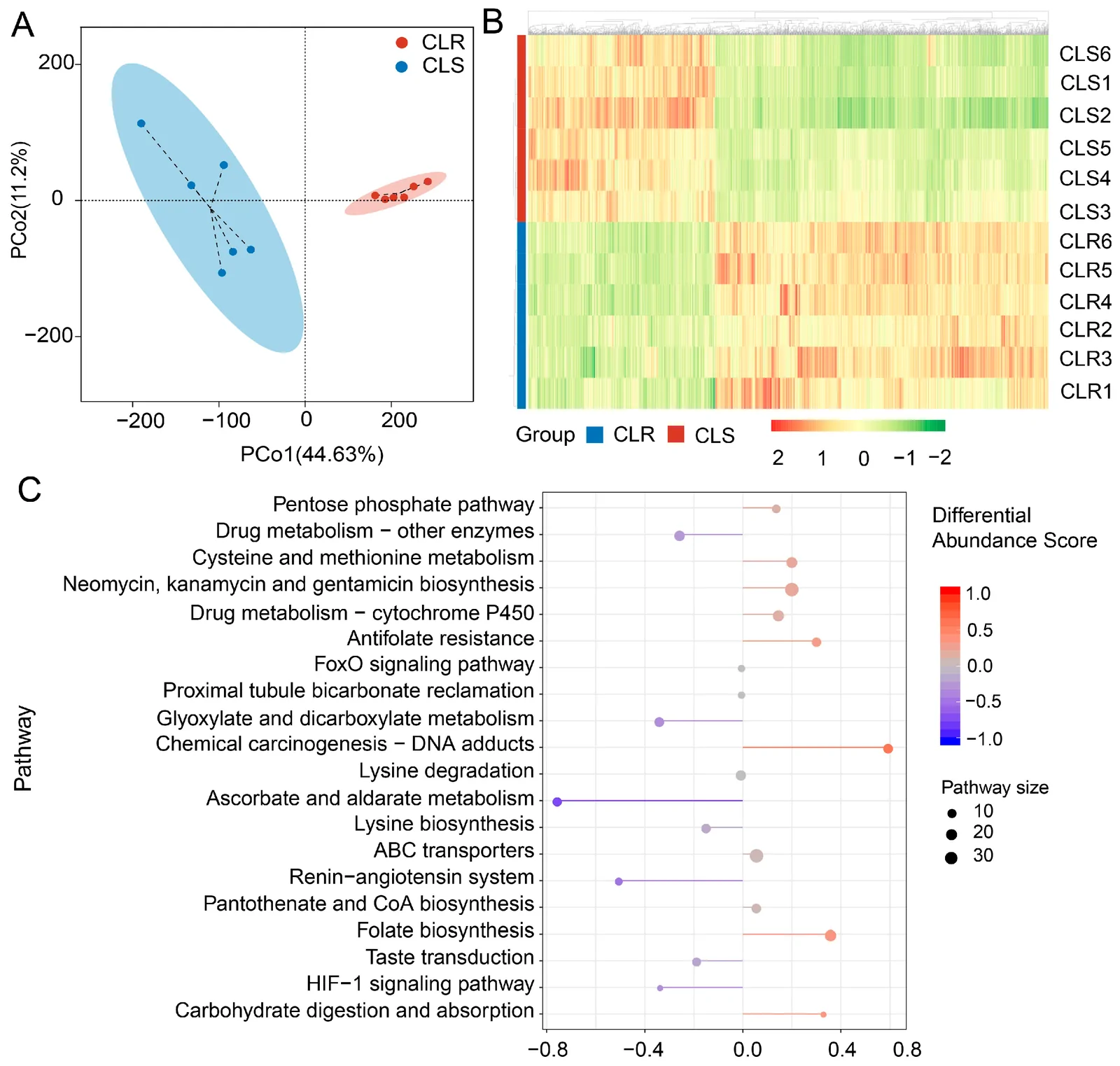
Global metabolomic differences between clothianidin-resistant (CLR) and susceptible (CLS) strains of the brown planthopper, *Nilaparvata lugens.* (**A**) Principal coordinate analysis (PCoA) of metabolite profiles. Each point represents one biological replicate (*n* = 6 per strain); PCo1 and PCo2 explain 44.63% and 11.20% of the total variation, respectively. (**B**) Hierarchical clustering heatmap of differential metabolites. Columns represent biological replicates and rows represent metabolites. Colors show row-standardized relative abundance (Z scores), ranging from −2 (green, lower relative abundance) to 2 (red, higher relative abundance). (**C**) Differential-abundance scores for significantly enriched KEGG pathways. Scores were calculated as (number of upregulated metabolites—number of downregulated metabolites)/(total number of differential metabolites in the pathway) and range from −1 (blue, predominantly downregulated in CLR relative to CLS) to 1 (red, predominantly upregulated). Dot size represents the number of differential metabolites assigned to each pathway (10–30).

To characterize metabolic alterations associated with resistance, we first compared the metabolite profiles of the resistant strain CLR and the susceptible strain CLS using widely targeted metabolomics. Principal coordinate analysis (PCoA) clearly separated CLR from CLS, with PCo1 and PCo2 explaining 44.63% and 11.20% of the total variation, respectively (55.83% combined), indicating substantial metabolic divergence between the two strains (Figure 1A). The six biological replicates within each strain clustered closely, supporting the robustness of the dataset. Consistently, hierarchical clustering also divided CLR and CLS into two distinct groups and revealed pronounced differences in metabolite accumulation patterns between the resistant and susceptible backgrounds (Figure 1B). Together, these results indicate that resistance in CLR is accompanied by a stable and global shift in metabolic state.

Pathway-level differential abundance analysis further showed that the altered metabolites were enriched in several pathways related to detoxification and intermediary metabolism, including the pentose phosphate pathway, drug metabolism by cytochrome P450, drug metabolism by other enzymes, ABC transporters, cysteine and methionine metabolism, glyoxylate and dicarboxylate metabolism, and ascorbate and aldarate metabolism (Figure 1C). These findings suggest that the resistant phenotype is associated not only with detoxification-related changes but also with broader remodeling of core metabolic processes.

### 3.2. Differential Metabolites Are Mainly Enriched in Central Carbon Metabolism and Detoxification-Related Pathways

**Figure 2:**
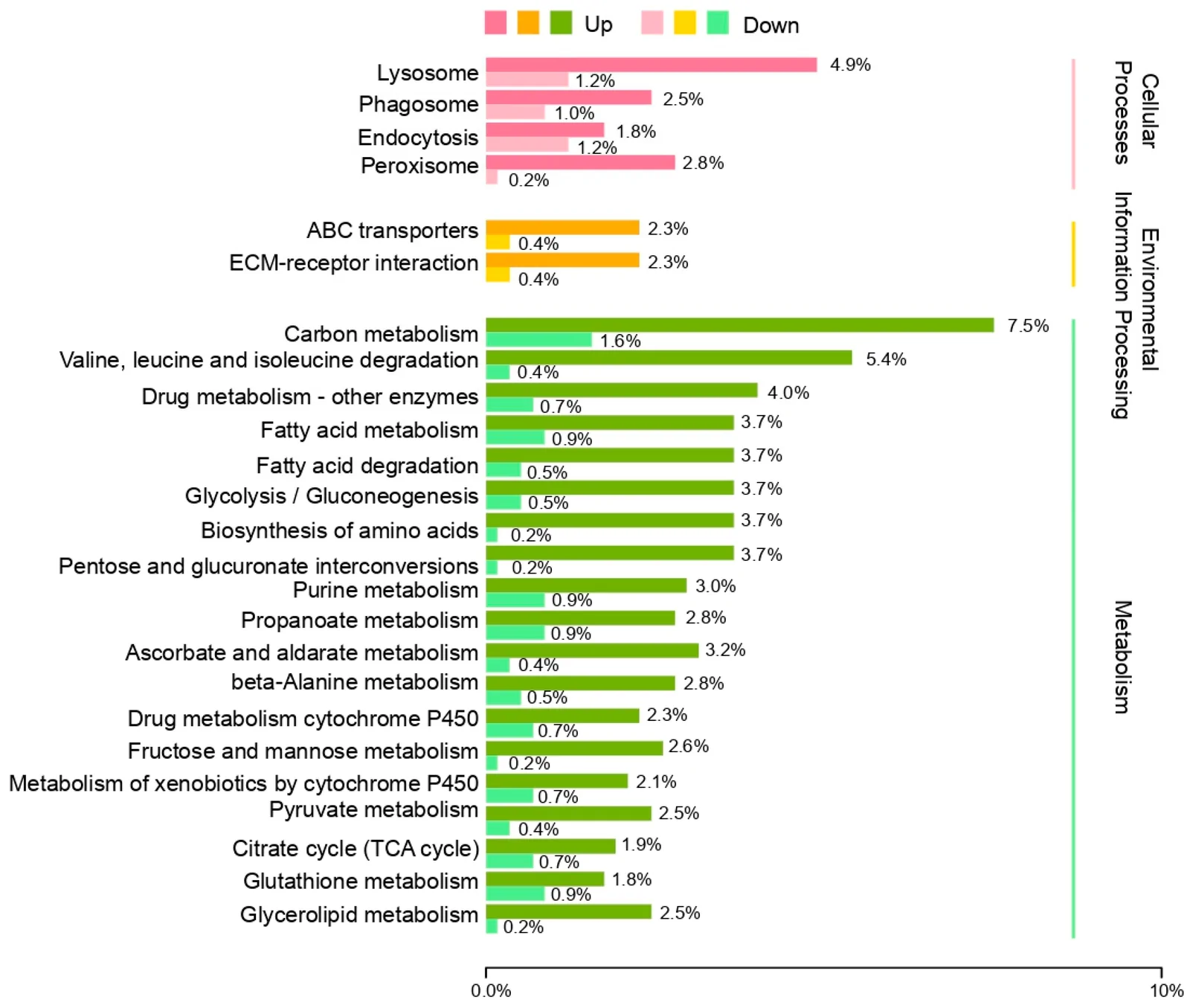
KEGG classification of differential metabolites between clothianidin-resistant (CLR) and susceptible (CLS) strains of the brown planthopper, *Nilaparvata lugens*. Differential metabolites were grouped into cellular process, environmental information processing, and metabolism categories. Bars represent the proportions of upregulated and downregulated metabolites within each pathway.

To further define the metabolic pathways altered in CLR, we classified differential metabolites based on KEGG annotations. Most differential metabolites were assigned to metabolism-related pathways, although some also fell into cellular process and environmental information processing categories (Figure 2). Among these, carbon metabolism showed the strongest enrichment, followed by glycolysis/gluconeogenesis, biosynthesis of amino acids, pentose and glucuronate interconversions, fructose and mannose metabolism, pyruvate metabolism, citrate cycle (TCA cycle), glutathione metabolism, metabolism of xenobiotics by cytochrome P450, and ABC transporters (Figure 2). The repeated appearance of these pathways suggests that resistant insects may reprogram energy and carbohydrate metabolism to support detoxification and physiological adaptation.

### 3.3. Integrated Transcriptome–Metabolome Analysis Identifies Key Resistance-Associated Metabolic Modules

**Figure 3:**
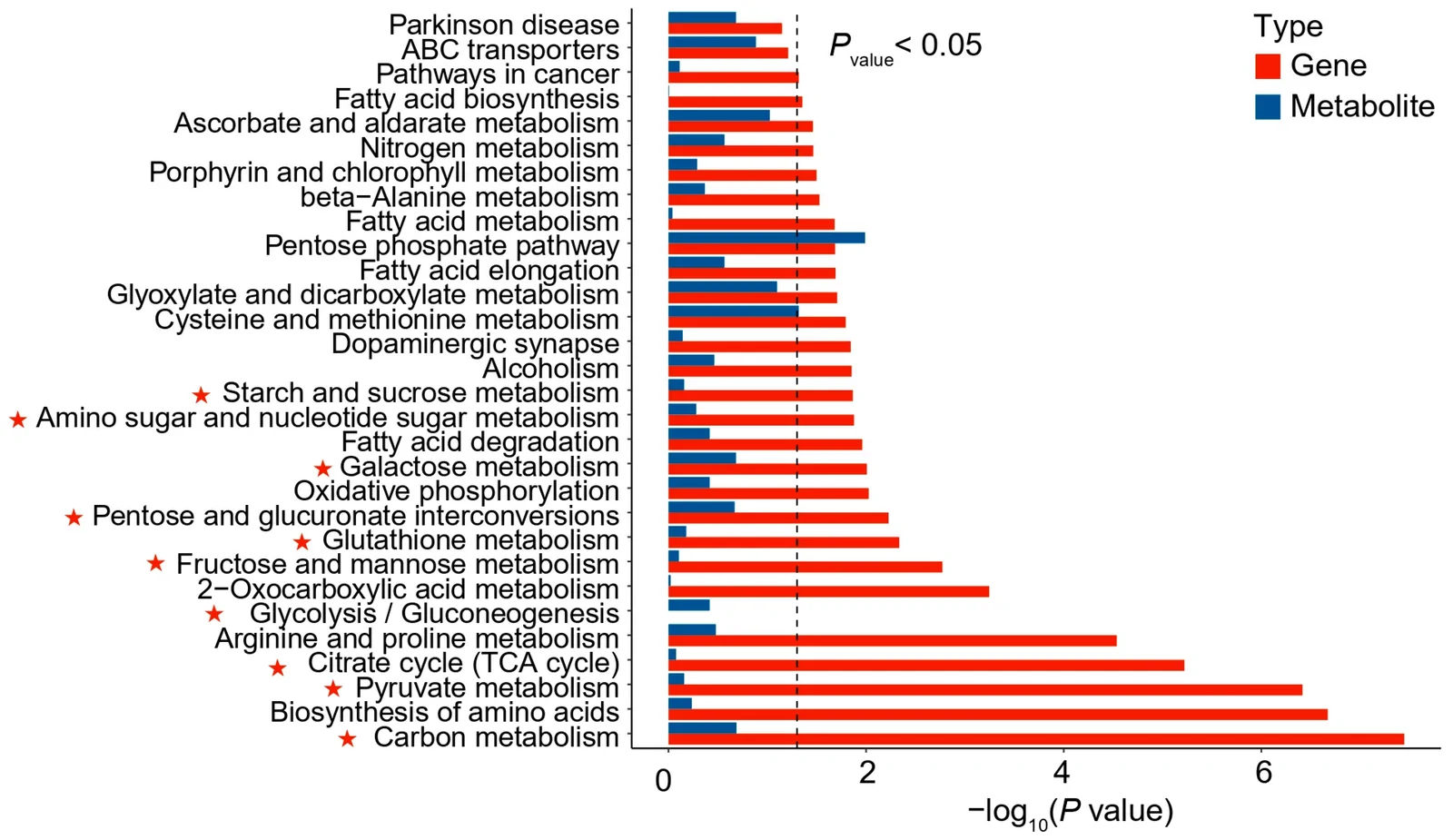
Integrated KEGG enrichment analysis of differentially expressed genes and differential metabolites in clothianidin-resistant and susceptible strains of the brown planthopper, *Nilaparvata lugens*. The *x*-axis shows enrichment significance as −log10 (*p* value). Red bars indicate gene enrichment and blue bars indicate metabolite enrichment. Only pathways with *p* < 0.05 are shown. The asterisks indicate pathways related to sugar metabolism and energy metabolism.

To integrate metabolic and transcriptional changes, differentially expressed genes and differential metabolites were jointly mapped to KEGG pathways. This combined analysis identified carbon metabolism as the most significantly enriched pathway, followed by biosynthesis of amino acids, pyruvate metabolism, citrate cycle (TCA cycle), glycolysis/gluconeogenesis, fructose and mannose metabolism, glutathione metabolism, and pentose and glucuronate interconversions (Figure 3). These recurrently enriched pathways point to coordinated changes in central carbon flux, organic acid metabolism, and reducing-equivalent supply in the resistant strain.

**Figure 4:**
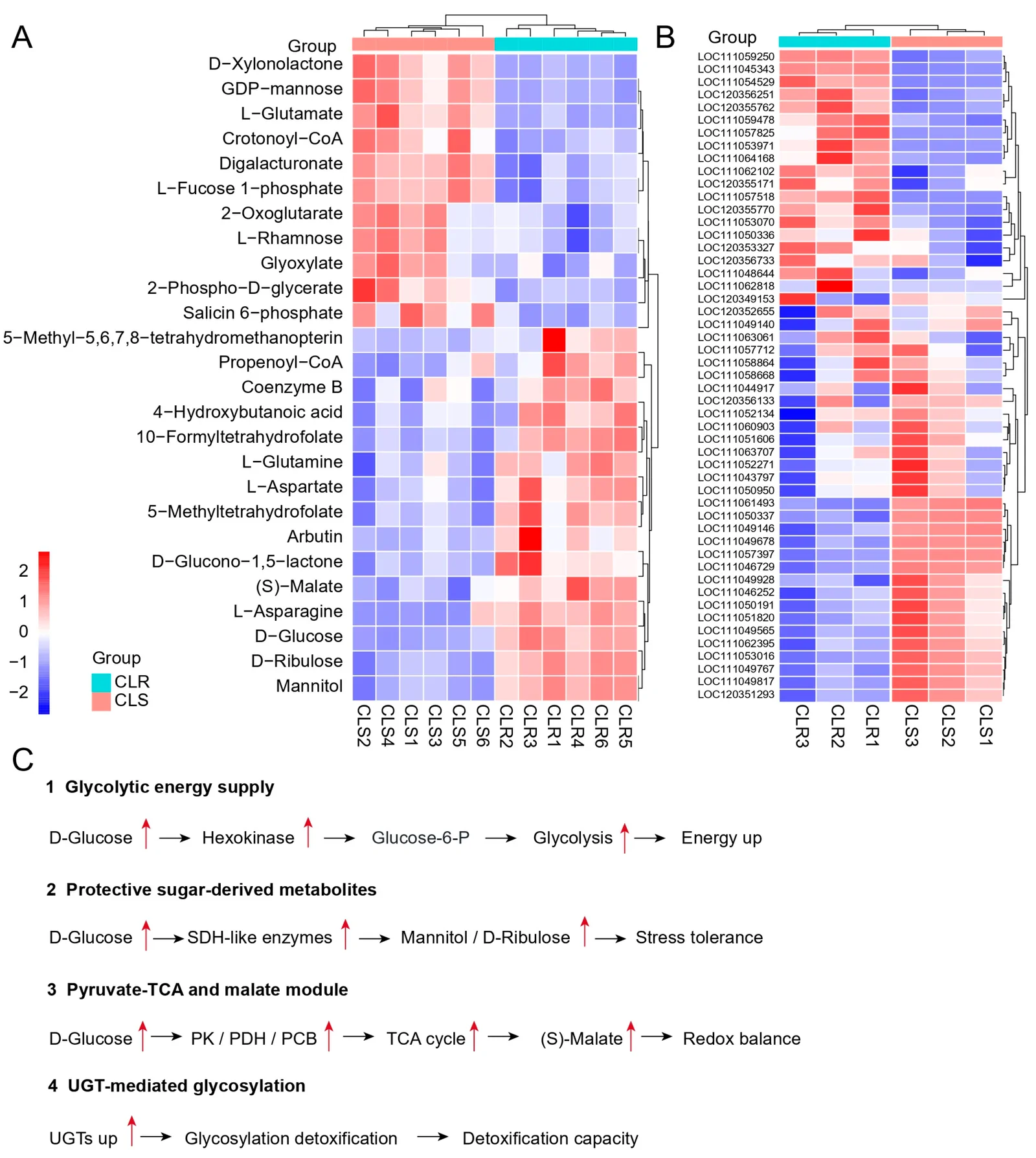
Resistance-associated metabolic modules in clothianidin-resistant (CLR) and susceptible (CLS) strains of the brown planthopper, *Nilaparvata lugens*. (**A**) Hierarchical clustering heatmap of representative metabolites in the key metabolic pathways. (**B**) Hierarchical clustering heatmap of representative differentially expressed genes in the same pathways. In (**A**) and (**B**), columns represent biological replicates and rows represent metabolites or genes, respectively. Colors indicate row-standardized relative abundance or expression (Z scores), ranging from −2 (blue, lower relative level) to 2 (red, higher relative level); cyan and salmon bars denote CLR and CLS samples, respectively. (**C**) Proposed functional model summarizing four resistance-associated modules: glycolytic energy supply, protective sugar-derived metabolites, pyruvate–TCA–malate metabolism, and UGT-mediated glycosylation. Red upward arrows indicate increased metabolite abundance, gene expression, or inferred pathway activity in CLR relative to CLS.

To visualize these relationships in greater detail, we constructed a gene-metabolite-pathway interaction network. The full supplementary network showed that numerous altered genes and metabolites converged on a limited number of core pathways, particularly carbon metabolism, glycolysis/gluconeogenesis, fructose and mannose metabolism, citrate cycle (TCA cycle), and pentose and glucuronate interconversions (Figure S1). Representative metabolites in these modules included D-glucose, 2-phospho-D-glycerate, 2-oxoglutarate, (S)-malate, D-ribulose, mannitol, arbutin, and salicin 6-phosphate (Figure 4A). Representative genes from the same pathways also showed clear strain-specific clustering (Figure 4B). We summarized these interactions into four major functional modules: glycolytic energy supply, protective sugar-derived metabolites, pyruvate-TCA and malate metabolism, and UGT-mediated glycosylation (Figure 4C). These results suggest that resistance in CLR is associated with coordinated remodelling of energy generation, stress-protective carbohydrate metabolism, organic acid turnover, and glycosylation-related detoxification.

### 3.4. qRT-PCR Validation Identifies Common Upregulated Candidate Genes in Two Resistant Backgrounds

**Figure 5:**
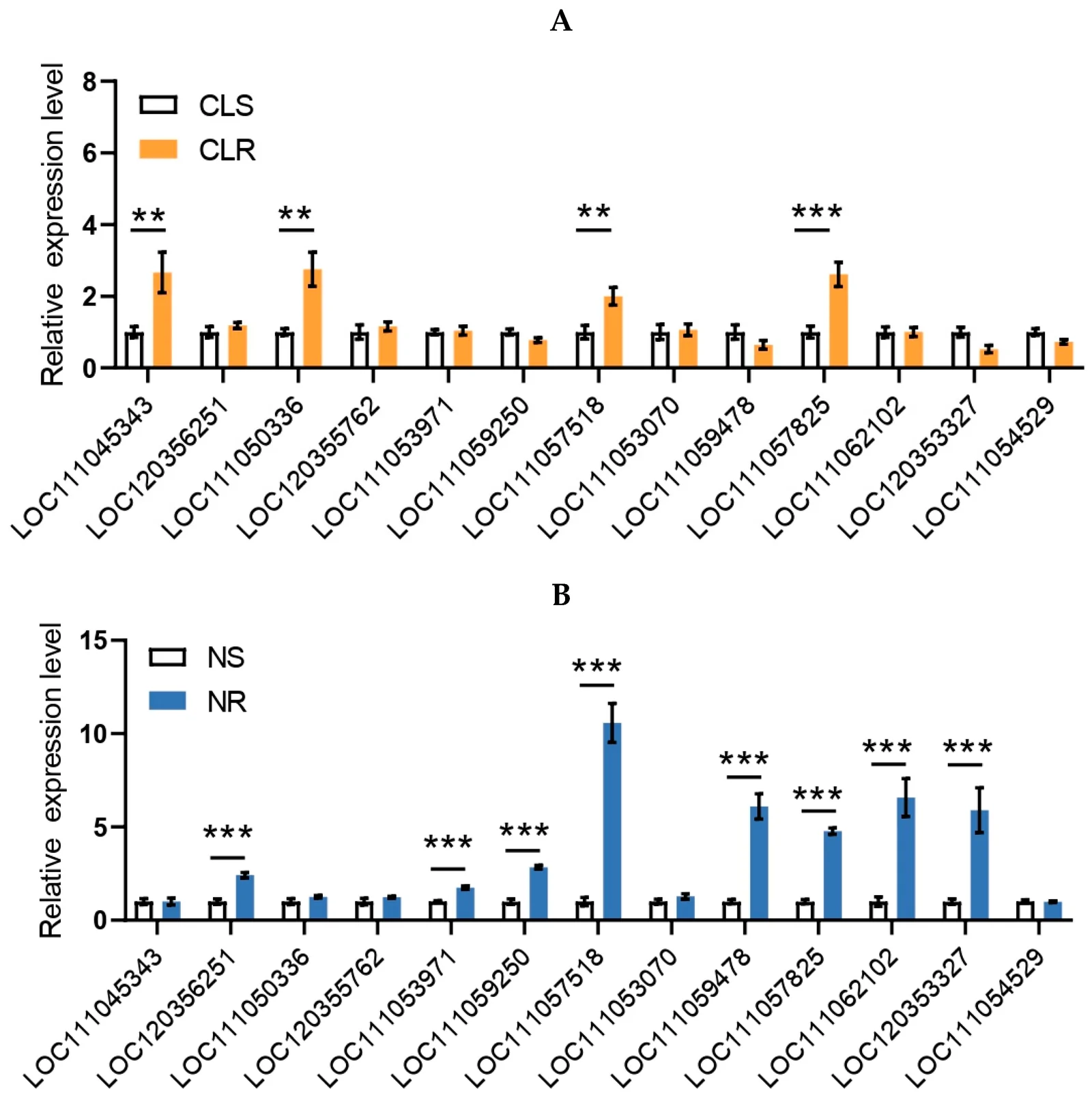
qRT-PCR validation of candidate genes in two neonicotinoid-resistant backgrounds of the brown planthopper, *Nilaparvata lugens.* (**A**) Relative expression levels of 13 candidate genes in the clothianidin-resistant strain CLR and the susceptible strain CLS. (**B**) Relative expression levels of the same 13 candidate genes in the nitenpyram-resistant strain NR and the susceptible strain NS. Relative transcript levels were determined by qRT-PCR and calculated using the 2^−ΔΔCt^ method. Data are presented as mean ± SEM. Asterisks indicate significant differences between resistant and susceptible strains (** *p* < 0.01 and *** *p* < 0.001).

Based on the integrated omics analysis, 13 candidate genes from the major enriched pathways were selected for expression validation. In the clothianidin-resistant strain CLR, qRT-PCR confirmed that several of these genes were differentially expressed relative to CLS, with multiple candidates showing significant upregulation in the resistant background (Figure 5A). To determine whether these candidates also responded in another neonicotinoid-resistant background, we further examined their expression in the nitenpyram-resistant strain NR and its susceptible counterpart NS. Several genes were strongly upregulated in NR relative to NS (Figure 5B).

Among the 13 candidates, two genes, *NADP* (LOC111057518) and *UGT2* (LOC111057825), were consistently upregulated in both resistant backgrounds (Figure 5A,B). Their shared induction in CLR and NR suggested that they may represent common resistance-associated targets across different neonicotinoid-resistant strains. Therefore, these two genes were selected for subsequent functional validation by RNA interference.

### 3.5. RNAi Validation Supports the Roles of NADP and UGT2 in Neonicotinoid Resistance Maintenance

To confirm the effectiveness of RNA interference prior to bioassays, transcript levels of *NADP* and *UGT2* were quantified after dsRNA treatment. Injection of ds*NADP* and ds*UGT2* markedly reduced the expression of their corresponding target genes relative to the ds*EGFP* control, indicating that both genes were efficiently silenced under the experimental conditions (Figure S2). These results established the basis for evaluating the effects of gene knockdown on insecticide susceptibility.

To determine whether *NADP* and *UGT2* functionally contribute to resistance, we compared insecticide susceptibility after RNAi treatment. Silencing of *NADP* significantly reduced the LC_50_ of nitenpyram from 90.06 mg/L in the ds*EGFP* group to 49.03 mg/L in the ds*NADP* group, corresponding to an LCR_50_ of 1.84 (Table 1). Similarly, the LC_50_ of clothianidin decreased from 291.77 mg/L to 161.19 mg/L after *NADP* knockdown, yielding an LCR_50_ of approximately 1.81 (Table 1). These data indicate that suppression of *NADP* partially restored susceptibility to both neonicotinoids.

Consistent with this pattern, silencing of *UGT2* also significantly enhanced insecticide susceptibility. After ds*UGT2* injection, the LC_50_ of nitenpyram declined from 98.15 mg/L in the ds*EGFP* group to 52.01 mg/L, corresponding to an LCR_50_ of 1.89 (Table 2). For clothianidin, the LC_50_ decreased from 299.33 mg/L to 163.99 mg/L after *UGT2* knockdown, with an LCR_50_ of 1.83 (Table 2). Taken together, these results show that both *NADP* and *UGT2* contribute to resistance maintenance in brown planthopper and support the conclusion that the resistance phenotype is linked to broader metabolic reprogramming rather than to isolated single-gene changes alone.

## 4. Discussion

In this study, we found that the clothianidin-resistant strain CLR and the susceptible strain CLS had markedly different metabolic profiles. The altered metabolites were concentrated in central carbon metabolism, glutathione metabolism, xenobiotic metabolism, ABC transport, and glycosylation. Joint transcriptome–metabolome analysis further organized these changes into four interconnected modules: glycolytic energy supply, protective sugar-derived metabolites, pyruvate–TCA–malate metabolism, and UGT-mediated glycosylation. *NADP* and *UGT2* were upregulated in both clothianidin- and nitenpyram-resistant backgrounds, and silencing either gene increased susceptibility to both insecticides. Previous studies have established that *CYP6ER1* contributes to resistance to several neonicotinoids in *N. lugens*, although its relative importance varies among compounds and resistance backgrounds [11,29,30,35,36,37]. Together with these earlier findings, our results indicate that resistance maintenance depends not only on elevated detoxification-enzyme expression but also on the metabolic supply of energy, reducing equivalents, and conjugation substrates.

The concurrent enrichment of carbon metabolism, glycolysis/gluconeogenesis, the TCA cycle, and the pentose phosphate pathway with P450-mediated xenobiotic metabolism, glutathione metabolism, and ABC transport is consistent with the biochemical demands of detoxification. P450 monooxygenase reactions require electrons delivered from NADPH through NADPH-cytochrome P450 reductase; GST reactions depend on reduced glutathione; UGT reactions consume activated UDP-sugars; and some detoxification products are exported by ATP-dependent ABC transporters [9,10,12,13,15,16]. Glycolysis and the TCA cycle sustain ATP production, whereas the pentose phosphate pathway and mitochondrial NADP-dependent dehydrogenases contribute to NADPH generation and glutathione recycling. The resistant strain may therefore redirect carbon flux to meet the energetic and cofactor requirements of P450s, GSTs, UGTs, and transport systems. In other insects, P450s that directly metabolize insecticides likewise depend on sufficient electron supply and a suitable intracellular redox environment [24,25]. Conversely, increased P450 turnover consumes NADPH and can generate reactive oxygen species through catalytic uncoupling. ROS-responsive CncC, JNK-ERK, and AP-1 pathways can in turn regulate P450 transcription [38,39,40], while circadian control of P450 expression and insecticide susceptibility further demonstrates that detoxification capacity is linked to the broader physiological state [41]. High temperature or antibiotic treatment disrupts symbiont-dependent detoxification in *N. lugens* and consequently increases insecticide susceptibility, providing additional evidence that metabolic and physiological conditions constrain resistance [31,42,43]. Thus, the coordinated alteration of central metabolism and detoxification pathways in our data is consistent with metabolic adaptation to the sustained cost of detoxification. Because metabolite abundance does not directly measure flux, this interpretation should be tested across additional resistant strains by quantifying sugars, organic acids, ATP, *NADPH*/*NADP*+, GSH/GSSG, and UDP-sugars, together with stable-isotope tracing of carbon redistribution.

Among the 13 candidate genes selected from the integrated analysis, *NADP* was upregulated in both clothianidin- and nitenpyram-resistant backgrounds, and its silencing increased susceptibility to both insecticides, supporting a functional association with resistance maintenance. NCBI annotates *NADP* as mitochondrial *NADP*-dependent isocitrate dehydrogenase rather than respiratory-chain NADH dehydrogenase; we therefore provisionally refer to it as *NADP*. This enzyme links the TCA cycle to mitochondrial *NADPH* metabolism by catalysing the conversion of isocitrate to α-ketoglutarate and contributing to glutathione reduction and mitochondrial redox homeostasis [44]. In light of the concurrent enrichment of the TCA cycle, glutathione metabolism, and xenobiotic metabolism, increased *NADP* expression could support P450 electron transfer and cellular stress tolerance by sustaining mitochondrial *NADPH* production, glutathione recycling, and ROS buffering [16,44]. The partial, rather than complete, restoration of susceptibility after knockdown is also consistent with resistance being maintained by multiple metabolic nodes. Measurements of enzyme activity, the isocitrate/α-ketoglutarate ratio, *NADPH*/*NADP*+, ROS, and GSH/GSSG would directly test this mechanism.

The expression and functional data for *UGT2* closely matched the UGT-mediated glycosylation module identified by the joint analysis. Insect UGTs use UDP-sugars to glycosylate xenobiotics or endogenous metabolites, thereby altering substrate activity, water solubility, and excretion [14,20]. UGT overexpression has also been linked to clothianidin resistance in *N. lugens* [45]. In our study, pathways related to pentose and glucuronate interconversions were altered, *UGT2* was consistently upregulated in two resistant backgrounds, and its silencing increased susceptibility to both clothianidin and nitenpyram. These observations support a model in which carbohydrate metabolism is adjusted to increase UDP-sugar availability and thereby sustain *UGT2*-mediated conjugation. *UGT2* could act directly on the insecticides or their primary metabolites, or it could modify endogenous metabolites that influence redox balance, hormonal signalling, or other detoxification pathways. Its direct substrates remain unresolved and should be identified by heterologous expression, substrate-turnover assays, and LC-MS/MS analysis of glycosylated products.

The altered central metabolic pathways and the RNAi phenotypes of the two candidate genes also point to possible resistance-intervention strategies. If resistant insects depend on elevated ATP and *NADPH* supply, inhibition of selected energy-producing or reducing-equivalent-generating steps could weaken sustained P450 activity and increase the efficacy of existing insecticides. Because central metabolism is highly conserved, broad-spectrum metabolic inhibitors are better suited initially as mechanistic probes; only insect-selective or tissue-enriched metabolic targets would be appropriate for further development. Nanomaterial-based synergistic approaches have already shown that interference with resistance-associated physiological processes can improve insecticide performance [46]. The sensitization caused by *NADP* and *UGT2* silencing also identifies both genes as potential targets for nucleic-acid-based synergists. Although oral RNAi in *N. lugens* is limited by nuclease degradation and tissue-delivery efficiency [47], nanoparticle-mediated fusion-dsRNA delivery and co-delivery of dsRNA with insecticides have demonstrated practical routes for improving resistance management [48,49].

In conclusion, our results link neonicotinoid resistance in *N. lugens* to coordinated remodelling of central metabolism and detoxification. Central carbon metabolism supplies ATP, NADPH, glutathione, and sugar donors to P450, GST, UGT, and ABC transport systems, whereas the energetic and redox burden imposed by detoxification may feed back on detoxification gene expression. *NADP* and *UGT2* connect this metabolic support network to *NADPH*/redox homeostasis and glycosylation, respectively, as supported by their shared upregulation across resistance backgrounds and the sensitization produced by RNAi. Metabolic resistance in *N. lugens* is therefore better understood as a coordinated adaptation between resource supply and xenobiotic clearance than as the isolated overexpression of individual detoxification genes. This framework broadens the metabolic basis of neonicotinoid resistance and provides a rationale for identifying synergistic targets within the metabolic support network.

## Figures and Tables

**Table 1:** Clothianidin and Nitenpyram LC_50_ against *N. lugens* after *dsNADP* injection.

**Insecticide**	**Treated**	**LC_50_ (95% CI ^a^) mg/L**	**Slope (SE)**	**χ2 (df)**	**LCR_50_ (95% CI ^b^)**
nitenpyram	ds*EGFP*	90.06 (67.25–119.94)	1.65 (0.25)	0.76 (3)	-
	ds*NADP*	49.03 (35.13–63.76)	1.89 (0.27)	1.98 (3)	1.84 (1.23–2.75) *
clothianidin	ds*EGFP*	291.77 (211.05–407.71)	1.44 (0.24)	0.56 (3)	-
	ds*NADP*	161.19 (118.60–206.80)	2.03 (0.28)	1.79 (3)	1.81 (1.19–2.75) *

^a^ LC_50_, median lethal concentration estimated by probit analysis; CI, confidence interval; SE, standard error of the slope; df, degrees of freedom. ^b^ LCR_50_, median lethal dose ratio, was calculated as the LC_50_ of the ds*EGFP* group divided by that of the target-gene dsRNA group. An asterisk indicates a significant difference from the ds*EGFP* control because the 95% CI of the LCR_50_ does not include 1.

**Table 2:** Clothianidin and Nitenpyram LC_50_ against *N. lugens* after *dsUGT2* injection.

**Insecticide**	**Treated**	**LC_50_ (95% CI ^a^) mg/L**	**Slope (SE)**	**χ2 (df)**	**LCR_50_ (95% CI ^b^)**
nitenpyram	ds*EGFP*	98.15 (69.39–137.59)	1.50 (0.25)	0.24 (3)	-
	ds*UGT2*	52.01 (38.52–66.37)	2.08 (0.29)	1.16 (3)	1.89 (1.24–2.88) *
clothianidin	ds*EGFP*	299.33 (213.40–413.08)	1.59 (0.26)	0.37 (3)	-
	ds*UGT2*	163.99 (125.07–206.51)	2.05 (0.27)	1.86 (3)	1.83 (1.22–2.73) *

^a^ LC_50_, median lethal concentration estimated by probit analysis; CI, confidence interval; SE, standard error of the slope; df, degrees of freedom. ^b^ LCR_50_, median lethal dose ratio, was calculated as the LC_50_ of the ds*EGFP* group divided by that of the target-gene dsRNA group. An asterisk indicates a significant difference from the ds*EGFP* control because the 95% CI of the LCR_50_ does not include 1.

## Data Availability

The data supporting the findings of this study are provided in the article and its Supporting Information. Additional raw data are available from the corresponding author upon reasonable request.

## Supplementary Materials

The following supporting information can be downloaded at: https://www.mdpi.com/article/10.3390/insects17090884/s1, Figure S1. Full gene-metabolite-pathway interaction network derived from the integrated transcriptomic and metabolomic analyses. Blue nodes represent genes, orange nodes represent metabolites, and red nodes represent pathways. Gray edges indicate the associations between genes or metabolites and the corresponding pathways. The network summarizes the overall relationships among candidate genes, metabolites, and enriched metabolic pathways. Figure S2. RNAi knockdown efficiency of NADP and UGT2. Relative expression changes in NADP and UGT2 after dsRNA treatment. The dsEGFP treatment served as the control. Each treatment included three independent biological replicates and three technical replicates per biological sample. Data are presented as mean ± SEM and were analyzed using Student’s *t*-test. Asterisks indicate significant differences from the dsEGFP control (*** *p* < 0.001). Table S1. Primers used for PCR, RT-qPCR, and dsRNA synthesis in this study.
