# Supplementary material for: Metabolic Reprogramming Supports Neonicotinoid Resistance in the Brown Planthopper, Nilaparvata lugens
Source: Insects. 2026 Aug 24;17(9):884. doi: 10.3390/insects17090884 (PMC13607281; doi:10.3390/insects17090884)
Supplement: Supplementary file 1 [file insects-17-00884-s001.zip › Fig S1.pdf]

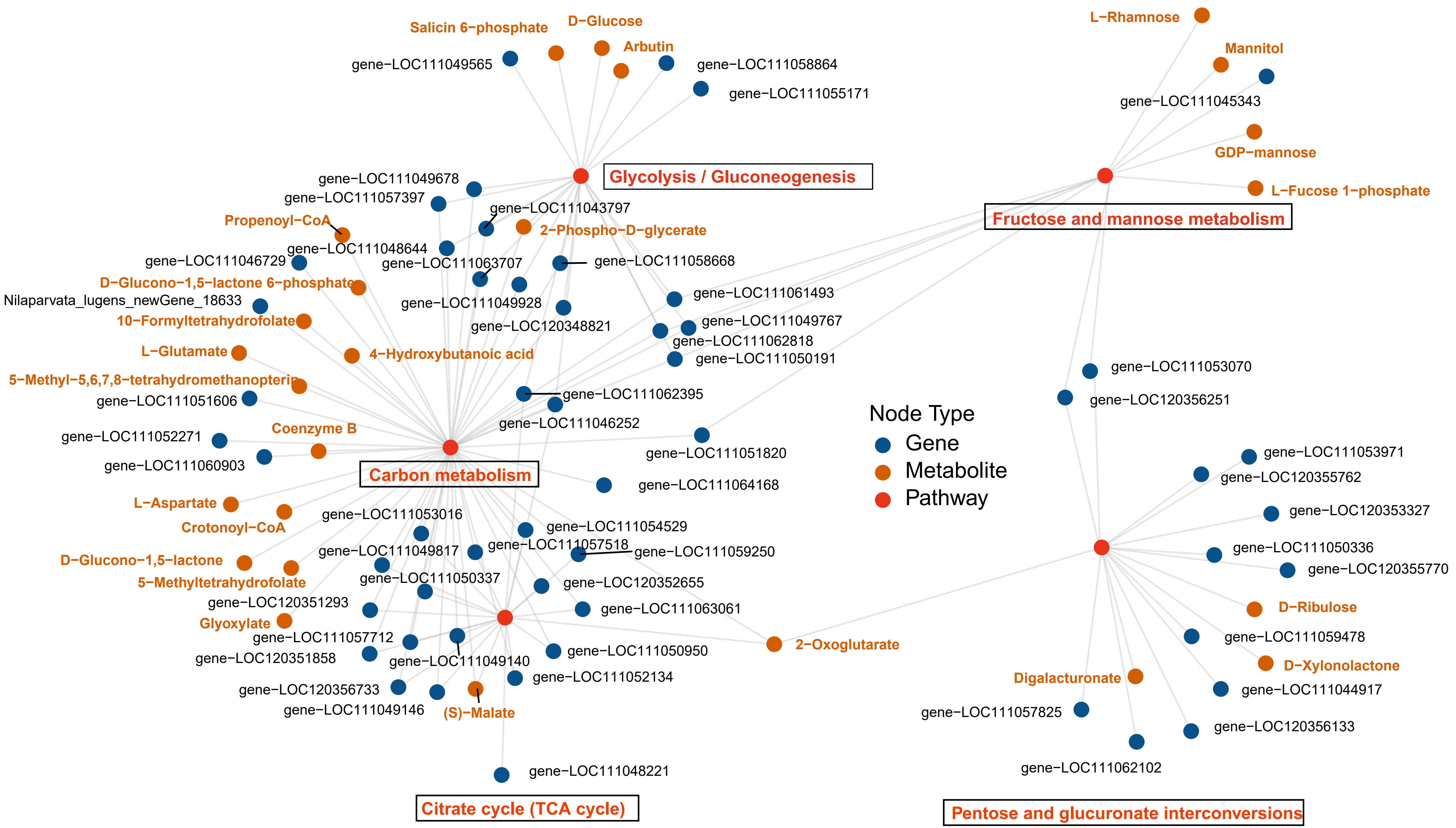
Glycolysis / Gluconeogenesis
Fructose and mannose metabolism
Carbon metabolism
Citrate cycle (TCA cycle)
Pentose and glucuronate interconversions
Node Type
Gene
Metabolite
Pathway
Salicin 6−phosphate
D−Glucose
Arbutin
gene−LOC111049565
gene−LOC111058864
gene−LOC111055171
L−Rhamnose
Mannitol
gene−LOC111045343
GDP−mannose
L−Fucose 1−phosphate
gene−LOC111049678
gene−LOC111057397
gene−LOC111043797
Propenoyl−CoA
2−Phospho−D−glycerate
gene−LOC111048644
gene−LOC111046729
gene−LOC111063707
gene−LOC111058668
D−Glucono−1,5−lactone 6−phosphate
Nilaparvata_lugens_newGene_18633
gene−LOC111049928
gene−LOC111061493
10−Formyltetrahydrofolate
gene−LOC120348821
gene−LOC111049767
gene−LOC111062818
L−Glutamate
4−Hydroxybutanoic acid
gene−LOC111050191
5−Methyl−5,6,7,8−tetrahydromethanopterin
gene−LOC111053070
gene−LOC111051606
gene−LOC111062395
gene−LOC120356251
gene−LOC111046252
Coenzyme B
gene−LOC111052271
gene−LOC111051820
gene−LOC111060903
gene−LOC111053971
gene−LOC120355762
gene−LOC111064168
L−Aspartate
gene−LOC111053016
Crotonoyl−CoA
gene−LOC120353327
gene−LOC111054529
gene−LOC111049817
gene−LOC111057518
gene−LOC111059250
D−Glucono−1,5−lactone
gene−LOC111050336
gene−LOC120355770
5−Methyltetrahydrofolate
gene−LOC111050337
gene−LOC120352655
gene−LOC120351293
gene−LOC111063061
D−Ribulose
Glyoxylate
gene−LOC111057712
2−Oxoglutarate
gene−LOC111059478
gene−LOC120351858
gene−LOC111049140
gene−LOC111050950
D−Xylonolactone
Digalacturonate
gene−LOC111052134
gene−LOC120356733
gene−LOC111044917
gene−LOC111049146
(S)−Malate
gene−LOC111057825
gene−LOC120356133
gene−LOC111048221
gene−LOC111062102
