# Supplementary material for: Metabolic Reprogramming Supports Neonicotinoid Resistance in the Brown Planthopper, Nilaparvata lugens
Source: Insects. 2026 Aug 24;17(9):884. doi: 10.3390/insects17090884 (PMC13607281; doi:10.3390/insects17090884)
Supplement: Supplementary file 1 [file insects-17-00884-s001.zip › Fig S2.pdf]

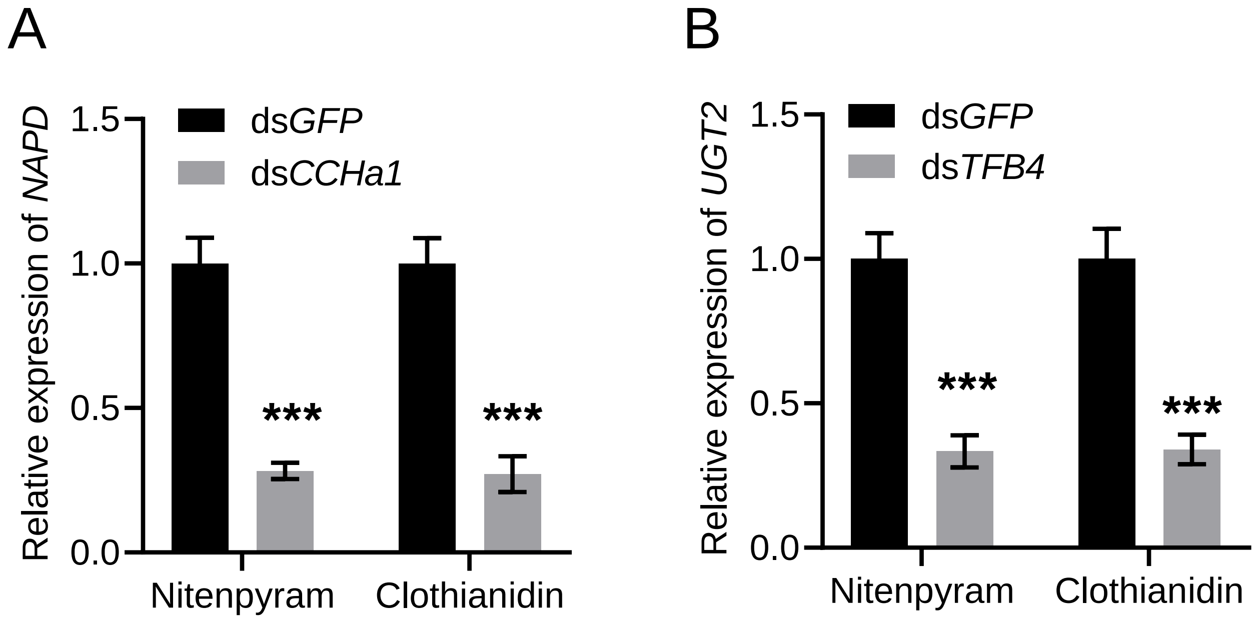

A
B
Relative expression of NAPD
1.5
1.0
0.5
0.0
dsGFP
dsCCHa1
***
***
Nitenpyram
Clothianidin
Relative expression of UGT2
1.5
1.0
0.5
0.0
dsGFP
dsTFB4
***
***
Nitenpyram
Clothianidin
