# Supplementary material for: Metabolic Reprogramming Supports Neonicotinoid Resistance in the Brown Planthopper, Nilaparvata lugens
Source: Insects. 2026 Aug 24;17(9):884. doi: 10.3390/insects17090884 (PMC13607281; doi:10.3390/insects17090884)
Supplement: Supplementary file 1 [file insects-17-00884-s001.zip › insects-4473104-supplementary.pdf]

Table S1 Primers used for PCR, RT-qPCR, and dsRNA synthesis in this study

Gene Symbol	Forward Primer (5'-3')	Reverse Primer (5'-3')
Nlβ-actin	CTGGACTTCGAGCAGGAAATGGC	CGACGTCGCACTTCATGATCGAG
Nl18s	CGCTACTACCGATTGAA	GGAAACCTTGTTACGACTT
LOC111045343	GATACTCTGGGAGCAGGAGATACG	CTTTGCTCCAGCCACCTTACAAGC
LOC120356251	CCACCAGGAACGGAGGAACTTTG	CAACTTCTCGGCACATTGCAGCCA
LOC111050336	ACGGCAGTTCTAAGTCAGCAGTGG	CGCATAGCATCGGCTTTGTTGGGT
LOC120355762	TAGGTCTGTTCACGAGGGTTACGG	GCCAACATATCTTTCAGCGGCGGA
LOC111053971	TGATTTCACGACGGGCTACCTGAG	CCTCTGCTCCGTCCATCCATTCTT
LOC111059250	TGCAGTTCCAGGCTTACTCTCTCG	CTGAGCCAGGTCTCCTACCACTTT
LOC111057518 *Isocitrate Dehydrogenase*	TGGACAGCCAACCTCTACGAATCC	CGCAAGATTCTTCCAGAGCGACAG
LOC111053070	AGTAGCAATAGAGCCAGGCGTCTC	CGACACAGATTGCCATCGTAGGGA
LOC111059478	TCGCCGATACCCATTGGCAGTAG	CTCTTGACGAGCAGCATTGGGCT
LOC111057825 UDP-glucosyltransferase 2	CCCCACTAAACGACAACCTCAACC	GAAGGCGTCTGCTCACCGAAATAC
LOC111062102	TGGAGTACGTCATCAGGCACAAGG	GAGAACGAATAGGACAGCAGCGAC
LOC120353327	TCTGTCCGTCGCTTACCCAATGAG	GCCTTGTTCAGCTCCGTCAATCCA
LOC111054529	TGTCCAGGCATTACTCACCCGAAG	GAGTAGATGGCTACGGAACGGATG
dsNADP	TAATACGACTCACTATAGTGGCGAGGCCAGGGTCAAAGAAT	TAATACGACTCACTATAGTGGTCTGTGCTCGTACCAGATGC
dsUGT2	TAATACGACTCACTATAGTGGGCAGGGCACTTCAGTTTCTG	TAATACGACTCACTATAGTGGGTGCGCTCCCTTATGTCGTA
dsEGFP	TAATACGACTCACTATAGGCCACAAGTTCAGCGTGTCCG	TAATACGACTCACTATAGGGGGTGGTCCCAATTCTCGTGGAAC
